# EARLY-LIFE ORIGINS OF LATER-LIFE HEALTH AMONG OLDER BRAZILIANS: DIFFERENCES IN ASSOCIATIONS BY HEALTH CONDITION

**DOI:** 10.1093/geroni/igad104.1247

**Published:** 2023-12-21

**Authors:** Mateo Farina

**Affiliations:** University of Texas at Austin, Austin, Texas, United States

## Abstract

**Background:**

A rich body of literature has linked poor childhood health to adverse adult health outcomes in later life. This association, however, has largely been studied in high-income countries. Less attention has been given to other country contexts where childhood exposures and adulthood socioeconomic conditions are vastly different. Brazil represents an important context due to the significant growth in older adult population, significant rise in life expectancy, and rapid industrialization that occurred since the 20th century. Method. Data come from the ELSI-Brazil Study, a nationally representative sample of older Brazilians. Logistic regression was used to predict the association between childhood conditions (socioeconomic adversity and poor health) and adult health conditions (heart conditions, heart failure, diabetes, and stroke).

**Results:**

Poor child health was positively associated with stroke, heart disease, and heart failure, but was not associated with diabetes or high blood pressure. Poor child socioeconomic status was associated with diabetes and stroke but not associated with heart conditions, heart failure, or high blood pressure.

**Discussion:**

The importance of childhood conditions for later life remained important in Brazil, but questions remain as to why certain conditions were found to be significant while others were not. Differences may stem from larger contextual changes such as the impact of globalization on food choices during adulthood for these cohorts. This study highlights the significance of contextual and conditional factors in comprehending the multifaceted dynamics underlying the association between early life conditions and later life health.

